# Executive function deficits mediate the relationship between employees' ADHD and job burnout

**DOI:** 10.3934/publichealth.2024015

**Published:** 2024-03-12

**Authors:** Yaara Turjeman-Levi, Guy Itzchakov, Batya Engel-Yeger

**Affiliations:** 1 Faculty of Social Welfare and Health Sciences, Department of Human Services, University of Haifa, 199 Aba Hushi Ave. Mount Carmel, Haifa, 3498838, Israel; 2 Faculty of Social Welfare and Health Sciences, Department of Occupational Therapy, University of Haifa, 199 Aba Hushi Ave. Mount Carmel, Haifa, 3498838, Israel

**Keywords:** attention-deficit/hyperactivity disorder (ADHD), employees, executive function deficits, work, job burnout

## Abstract

Adults with Attention-Deficit/Hyperactivity Disorder (ADHD) often face significant deficits in executive function and adverse work-related outcomes. This study aimed to explore the role of executive function deficits in job burnout of employees with ADHD. We hypothesized that employees with ADHD, relative to employees without ADHD, will experience higher levels of job burnout and deficits in executive function. We also hypothesized that the ADHD-job burnout relationship would be mediated through executive function deficits, specifically by self-management to time and self-organization/problem-solving. A field study with 171 employees provided support for the research hypotheses and mediation model in which the employees' ADHD-job burnout relationship was mediated through executive function deficits. Additional mediation analyses indicated that the specific executive function of self-management to time and self-organization/problem-solving mediated the effect of ADHD on job burnout and its facets. Specifically, for physical fatigue, the mediation was realized through self-management to time, and for emotional exhaustion and cognitive weariness, the mediation was significant through self-organization/problem-solving. The present findings shed light on the relevance of referring ADHD among employees, their vulnerability to job burnout, and the role of executive function deficits in job burnout of employees with ADHD.

## 1. Introduction

Attention-Deficit/Hyperactivity Disorder (hereafter ADHD), characterized by impairing levels of inattention, disorganization, and hyperactivity-impulsivity [1], is a neurodevelopmental disorder that often persists into adulthood, affecting a considerable proportion of individuals [2]–[6].

Despite its prevalence, adults with ADHD face various adverse work-related outcomes, including failure to meet self-standards and perceived potential [7], increased absenteeism [8], lower occupational status [9], reduced job stability [10], unemployment, and challenges in work performance [11]–[13]. Therefore, it is not surprising that adults with ADHD suffer from work-related health issues such as stress and sickness [14]. However, the exploration of specific mechanisms underlying these challenges has been limited.

The present work focuses on a specific adverse workplace outcome for employees with ADHD—job burnout. We propose that employees with ADHD will experience higher levels of job burnout compared to those without ADHD. Additionally, given the relationship between ADHD and deficits in executive function (hereafter EF) [15] and the predictive role of EF deficits in job burnout [16], we propose deficits in EF as a mechanism for this relationship.

We aim to elucidate the relationship between ADHD, EF deficits, and job burnout, providing the theoretical rationale for each link. This understanding may offer insights into addressing the negative impact of EF deficits on job burnout among employees with ADHD.

### 1.1. Adult ADHD and job burnout: Workplace challenges

Job burnout, defined as a psychological syndrome resulting from a negative affective state, stems from the continuous depletion of energetic coping resources due to chronic exposure to occupational stress [17],[18]. It encompasses symptoms of (a) *physical fatigue*—an extreme physical tiredness, (b) *emotional exhaustion*—a depletion of emotional resources, and (c) *cognitive weariness*—a sense of cognitive overload [17],[19],[20]. These symptoms carry substantial workplace repercussions, including job dissatisfaction [21],[22], unjustified absenteeism [22]–[25], poorer quality of care [26], professional mistakes [27],[28], and abandonment [22],[25],[29].

Job burnout and ADHD share associations with impairments in cognitive processes essential for goal-directed activities, such as planning, organizing, and regulating attention and memory [30],[31], which are manifested in difficulties at the workplace [32]. Moreover, ADHD adversely impacts adaptive coping and work process improvement, which are associated with reduced burnout [33]. To our present knowledge, the literature has largely ignored the relationship between ADHD and job burnout, with few exceptions [34].

Thus, in the present study, we aim to fill this gap. We propose that ADHD symptoms, reflecting work-related difficulties, may exacerbate the negative effects of common job stressors such as workload (due to lack of organization, short attention span, increased errors, and memory deficits), conflicted relationships, and values misalignment on the job (due to poor social skills and restlessness), and therefore may lead to a greater depletion of energetic coping resources, resulting in increased occupational stress and elevated burnout levels. Hence,

*Hypothesis*_1_: Employees with ADHD would report higher job burnout than employees without ADHD.

### 1.2. Executive function deficits of employees with ADHD

The core facets of EF are response inhibition, working memory, and shifting [35], which include (a) *Self-management to time*—planning and prioritizing tasks, (b) *Self-organization/problem-solving*—organizing tasks and strategic problem-solving, (c) *Self-restraint*— controlling impulsive behaviors, (d) *Self-motivation*—setting and achieving goals, and (e) *Self-Regulation of emotion*—managing and controlling emotional responses [36].

Deficits in EF exert a direct impact on fundamental abilities, limiting daily function [37], social interactions, academic performance [38], social activities [39], ineffective time management [31],[40]–[42], lack of organization [31],[43],[44], short attention span [31],[42],[43],[45], memory deficits [31],[44], increased errors [31],[44], restlessness [31],[43], and poor social skills [31],[40],[43],[44].

Therefore, deficits in EF manifest as a diverse range of issues, impacting various aspects of individuals' daily lives, including their experiences in the workplace. For instance, experiencing EF deficits may lead to struggling with time management, finding it challenging to organize schedules efficiently and complete tasks within set deadlines. Additionally, it might encounter problems in self-regulation of emotions, leading to difficulties in coping with stress or regulating reactions in various situations.

In the specific context of the workplace, employees with deficits in EF may experience difficulties in multitasking, setting priorities, and adapting to changing work demands. They might struggle with decision-making processes, leading to suboptimal choices in professional settings. Furthermore, deficits in EF can impact interpersonal relationships at work, affecting communication, collaboration, and conflict resolution.

Numerous empirical studies consistently associate deficits in EF facets with ADHD. A meta-analysis of the relationship between EF facets and ADHD (*N* = 83, *k* = 6703) found significant deficits across all EF dimensions in individuals with ADHD [15]. Additionally, adults with ADHD were found to exhibit deficits in EF [46],[47]. Therefore, we hypothesize that:

*Hypotheses _2_:* Employees with ADHD, relative to employees without ADHD, would report a higher level of deficits in each dimension of the EF: (a) *Self-management to time*, (b) *Self-organization/problem-solving*, (c) *Self-restraint*, (d) *Self-motivation*, and (e) *Self-Regulation of emotion*.

Furthermore, deficits in EF among employees with ADHD may serve as mediating factors for job burnout. This suggests a potential link between EF deficits and the negative impact on job burnout, providing additional insight into the challenges faced by employees with ADHD.

### 1.3. Executive function deficits mediating the link between employees' ADHD and job burnout

Deficits in EF, particularly experienced by employees with ADHD, present considerable challenges that may substantially contribute to job burnout. Ineffective time management and organizational skills can escalate work-related stress, leading to weariness, frustration, emotional exhaustion, and physical fatigue. A short attention span and memory deficits amplify the risk of errors, reduce motivation to fulfill job responsibilities, and hinder meeting deadlines, ultimately resulting in emotional and physical exhaustion. Additionally, poor social skills and difficulties in regulating emotions can adversely affect interpersonal relationships and the overall ability to work, further intensifying stress and physical fatigue. As these challenges accumulate in the work environment, employees with EF deficits, especially those with ADHD, may experience a depletion of energetic coping resources, a key component of job burnout [25]. The constant effort required to navigate these challenges in the work environment may contribute to emotional exhaustion, cognitive weariness, Physical fatigue, and overall heightened risk of burnout. Thus, the intricate interplay of these challenges underscores the potential for employees with EF deficits, particularly those with ADHD, to confront a heightened risk of job burnout.

Despite the workplace relevance of EF deficits, organizational research has largely neglected it. Recently, Chan et al. (2021) [35] mentioned this gap and highlighted the pivotal role of proper EF in essential managerial behaviors such as decision-making, planning and monitoring, problem-solving, negotiating, and innovating.

**Figure 1.**
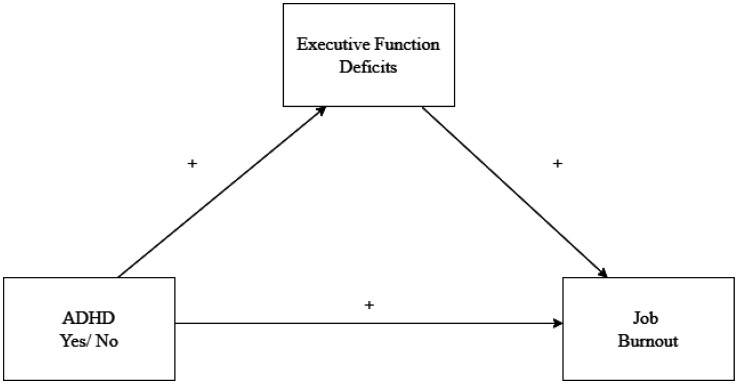
A theoretical model of the effect of ADHD on job burnout through executive function deficits.

Given that ADHD is associated with clinically significant impairments, which manifest as deficits in EF [15],[46],[48],[49], and deficits in EF, in turn, includes deficits in fundamental abilities that are highly relevant to the workplace, we propose that these negative work-related consequences of EF may lead to emotional and interpersonal stress at work, resulting in burnout. Figure 1 presents our theoretical mediation model, including the following hypothesis:

*Hypothesis*_3a_: The relationship between ADHD and job burnout would be mediated by EF.

Furthermore, previous literature regarding ADHD suggests that the deficits observed in time management and organizational skills, inherent in *self-management* and *self-organization/problem-solving* facets, may contribute to difficulties manifested in the workplace, such as inefficiencies and increased stress [31],[40]–[43]. Deficits in these specific EF can lead to challenges related to planning, organizing tasks, managing time efficiently, adhering to schedules, solving problems effectively, prioritizing tasks, and organizing work efficiently. These deficits can result in increased stress due to increased work demands, inefficient task completion, heightened frustration, and depletion of energetic coping resources, ultimately contributing to job burnout. Therefore, we propose that *self-management to time* and *self-organization/problem-solving* serve as mechanisms through which ADHD symptoms impact job burnout. Thus:

*Hypothesis*_3b_: *Self-management to time* and *Self-organization/problem-solving* would be the strongest mediators of the relationship between ADHD and job burnout.

## 2. Materials and methods

### 2.1. Participants & procedure

The study was conducted after obtaining approval from our University's Ethics Committee (approval number 225/21), and informed consent was obtained from all participants. Data collection occurred in 2021 amidst the COVID-19 pandemic. We recruited 209 participants through Prolific Academic (*M_age_* = 25.86, *SD* = 0.49, 59.51% female) following a-priori power analysis that indicated that this sample size provides a power of 0.95 to detect a moderate correlation, *r* = 0.25 [50]. Of this sample, 38 of the participants reported being “Out of work” or “Retired” and were subsequently excluded from the data analysis, aligning with the study's focus on employees. Therefore, the final sample size included 171 participants; 71 worked full-time, 74 worked part-time, 22 were Self-employed, and four were unidentified. The mean age was 26.45, *SD* = 0.49, mean seniority = 1.66 years, *SD* = 0.96, 59 participants had tenure and, 107 did not have tenure (five participants did not provide information regarding tenure), 60.48% were female. The participants worked in various professions, such as sales, office administration, and education.

### 2.2. Measures

#### 2.2.1. Adult ADHD

ADHD is known to be under-identified and under-treated in the adult population [51],[52]. Therefore, we did not rely on self-reports of clinical diagnoses but used the six-item Adult ADHD Self-Report Screening Scale for DSM-5 (hereafter ASRS-5) [53]. This scale has previously demonstrated the ability to detect the vast majority of cases in the general population with a threshold of 14 for the sum of the measure scores, with excellent psychometric properties [53], α *=* 0.63.

#### 2.2.2. Executive function deficits

We used the 20-item Barkley Deficits in Executive Functioning Scale – Short Form (hereafter BDEFS-SF) [54]. The BDEF-SF includes five subscales: *self-management to time*, *self-organization/problem-solving*, *self-restraint*, self-motivation, and *self-regulation of emotion*. The BDEF-SF includes four items for each of the five subscales and is widely used in adult studies (previous alpha range = 0.92 to 0.94) [36],[55],[56]. In the current study, reliabilities were as follows: for *self-management to time*, *α =* 0.84, for *self-organization/problem-solving*, *α* = 0.84, for *self-restraint*, *α = 0.80*, for *self-motivation*, *α =* 0.86, and for *self-regulation of emotion*, *α =* 0.87.

#### 2.2.3. Job burnout

We used the 14-item Shirom-Melamed Burnout Measure (hereafter SMBM) [57]. The SMBM includes three subscales: *physical fatigue*, *emotional exhaustion*, and *cognitive weariness*. SMBM is a widely applied instrument with excellent internal consistency (*α* > 0.92) [58],[59]. In the current study, the reliabilities were as follows: for the total SMBM, *α =* 0.94, for *physical fatigue*, *α =* 0.92, for *emotional exhaustion*, *α = 0.81*, and for *cognitive weariness*, *α =* 0.88.

## 3. Results

Table 1 presents the correlations among the variables. Table 2 presents the results of independent sample *t*-tests for employees with and without ADHD.

Based on the ASRS-5 screening results, with a threshold of 14 [53], our sample included 38 participants with ADHD and 133 without ADHD. The assumption of equal variances was assessed using Bartlett's and Levene's tests. Bartlett's test indicated no significant difference in variances between groups K^2^ (1) = 0.63, *p* = 0.43, supporting the equality of variances assumption. Levene's test also supported this finding, *F* (1,126) = 1.25, *p* = 0.45.

### 3.1. Main effects

#### 3.1.1. Job burnout

An independent t-test revealed a significant main effect of employees' ADHD on job burnout, *t*(62) = 6.34, *p* < 0.001, 95% *CI* [0.73, 1.51], *d* = 1.13. Consistent with H1, employees who identified themselves with ADHD (ASRS-5 sum score ≥ 14) reported higher job burnout (*M* = 5.05, *SD* = 1.05) compared to those without ADHD (*M* = 3.76, *SD* = 1.17). Moreover, as shown in Table 2, employees with ADHD, relative to employees without ADHD, reported higher levels of each dimension of job burnout.

#### 3.1.2. Executive function deficits

A strong and significant main effect of employees' ADHD was observed on overall EF deficits, *t*(62) = 7.51, *p* < 0.001, 95% *CI* [0.94, 1.73], *d* = 1.34. Employees identified with ADHD (ASRS-5 sum score ≥ 14) reported higher EF deficits (*M* = 2.65, *SD* = 0.50) compared to those without ADHD (*M* = 1.92, *SD* = 0.55). Moreover, as shown in Table 2, and consistent with H2a-e, employees with ADHD, relative to employees without ADHD, reported higher levels of deficits in each dimension of the EF.

**Table 1.** Descriptive statistics and correlations for study variables.

**Measure**	**N**	**Mean**	**SD**	**1**	**2**	**3**	**4**	**5**	**6**	**7**	**8**	**9**	**10**	**11**	**12**	**13**	**14**
1. ADHD (0 = No; 1 = Yes)	171	0.22	0.42	(-)													
2. ASRS-5	169	1.73	0.69	0.74**	(0.63)												
2. Executive Function Deficits: Total	164	2.08	0.62	0.49**	0.71**	(0.93)											
3. Self-management to time	169	2.66	0.80	0.46**	0.63**	0.77**	(0.84)										
4. Self-organization/problem-solving	168	2.00	0.80	0.37**	0.52**	0.76**	0.47**	(0.84)									
5. Self-restraint	170	1.83	0.72	0.40**	0.53**	0.79**	0.48**	0.60**	(0.80)								
6. Self-motivation	167	1.88	0.82	0.38**	0.58**	0.84**	0.61**	0.55**	0.58**	(0.86)							
7. Self-regulation of emotion	170	2.06	0.85	0.33**	0.52**	0.74**	0.47**	0.41**	0.50**	0.54**	(0.87)						
8. Burnout: Total	163	4.05	1.26	0.43**	0.55**	0.63**	0.54**	0.54**	0.47**	0.47**	0.43**	(0.94)					
9. Physical fatigue	169	4.50	1.42	0.36**	0.47**	0.49**	0.49**	0.35**	0.36**	0.36**	0.39**	0.87**	(0.92)				
10. Emotional exhaustion	168	4.12	1.53	0.37**	0.53**	0.59**	0.46**	0.51**	0.43**	0.51**	0.42**	0.91**	0.79**	(0.81)			
11. Cognitive weariness	169	3.72	1.47	0.37**	0.51**	0.60**	0.45**	0.60**	0.46**	0.44**	0.39**	0.89**	0.58**	0.77**	(0.88)		
12. Gender (1 = Male; 2 = Female)	167	1.60	0.49	0.01	0.03	0.01	0.02	0.03	-0.07	-0.09	0.12	0.09	0.19*	0.07	0.07	(-)	
13. Age	166	26.45	7.66	-0.14	-0.22**	-0.26**	-0.24**	-0.15	-0.16*	-0.20**	-0.24**	-0.25**	-0.27**	-0.22**	-0.19*	-0.08	(-)

Notes: Values in the diagonal are reliabilities; *p < 0.05. **p < 0.01.

**Table 2.** Comparison of burnout and executive function between adults with and without ADHD.

***Measure***	**No ADHD**		**ADHD**		***t*-value**	***df***	***p***	***d* [95% *CI*]**
	***M***	***SD***	***M***	***SD***				
**Burnout: Total**	3.76	1.17	5.05	1.05	6.34	62	< 0.001	1.13 [0.73, 1.51]
Physical fatigue	4.22	1.40	5.45	1.05	5.85	79	< 0.001	0.92 [0.55, 1.30]
Emotional exhaustion	3.82	1.46	5.19	1.27	5.60	65	< 0.001	0.96 [0.58, 1.34]
Cognitive weariness	3.43	1.33	4.75	1.47	4.92	54	< 0.001	0.97 [0.59, 1.35]
**Executive function deficits: Total**	1.92	0.55	2.65	0.50	7.51	62	< 0.001	1.34 [0.94, 1.73]
Self-management to time	2.46	0.74	3.34	0.57	7.75	78	< 0.001	1.25 [0.88, 1.61]
Self-organization/problem-solving	1.85	0.74	2.55	0.76	5.02	56	< 0.001	0.95 [0.57, 1.33]
Self-restraint	1.68	0.63	2.36	0.75	5.09	53	< 0.001	1.03 [0.65, 1.41]
Self-motivation	1.72	0.75	2.47	0.80	5.11	55	< 0.001	0.99 [0.61, 1.37]
Self-regulation of emotion	1.91	0.81	2.58	0.76	4.75	63	< 0.001	0.84 [0.47, 1.21]

Note: Cohen's d values indicate that employees with ADHD scored higher on the respective burnout or executive function scale.

### 3.2. Mediation analysis

**Figure 2.**
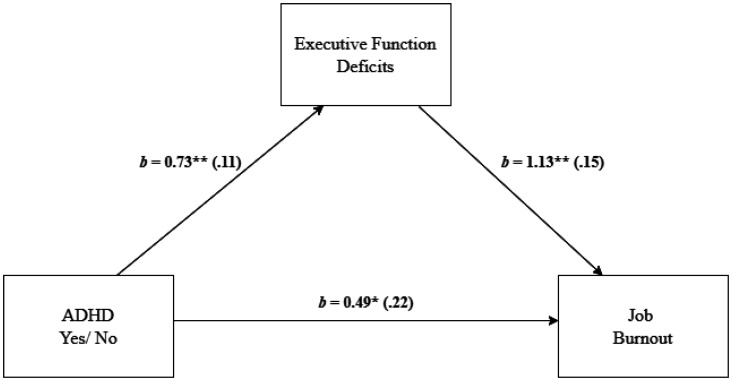
Mediation analysis for the relationship between employees' ADHD and job burnout, mediated by executive function deficits. Note: Standard errors are in parentheses. *p < 0.05, **p < 0.01.

To examine whether EF deficits explained the effect between employees' ADHD and job burnout, we conducted a mediation analysis using Model 4 in PROCESS [60]. As shown in Figure 2, EF deficits mediated the effect of employees' ADHD on job burnout, as indicated by a significant indirect effect, *b* = 0.83, *SE* = 0.16, 95% *CI* [0.55, 1.16], providing support for H3a. Namely, Employees with ADHD experienced more EF deficits than non-ADHD employees, and EF deficits were positively associated with job burnout. The direct effect was also significant, *b* = 0.49, *SE* = 0.22, 95% *CI* [0.07, 0.92].

**Figure 3.**
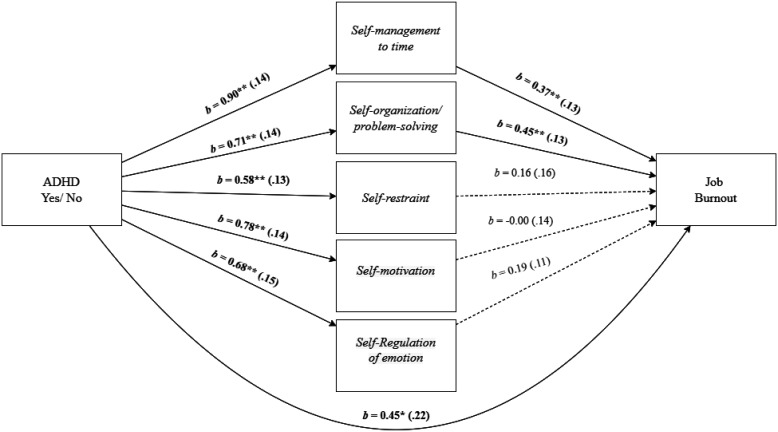
Mediation analysis for the relationship between employees' ADHD and job burnout, mediated by executive function deficits of self-management to time and self-organization/problem-solving. Note: Standard errors are in parentheses. *p < 0.05, **p < 0.01.

To examine whether employees' EF deficits, specifically in *Self-management to time* and *Self-organization/problem-solving*, mediated the effect between employees' ADHD and job burnout, we used Model 4 in PROCESS [60]. As shown in Figure 3, employees' EF deficits of *Self-management to time* and *Self-organization/problem-solving* mediated the effect of employees' ADHD and job burnout, as indicated by a significant total indirect effect, *b* = 0.87, *SE* = 0.18, 95% CI [0.55, 1.26], and specifically indicated by a significant indirect effect of *Self-management to time, b* = 0.33, *SE* = 0.16, 95% *CI* [0.03, 0.66], and *Self-organization/problem-solving, b* = 0.32, *SE* = 0.12, 95% *CI* [0.12, 0.58]. The direct effect remained significant, *b* = 0.45, *SE* = 0.22, 95% *CI* [0.01, 0.86].

### 3.3. Exploratory mediation analysis on each burnout subscale

#### 3.3.1. Physical fatigue

**Figure 4.**
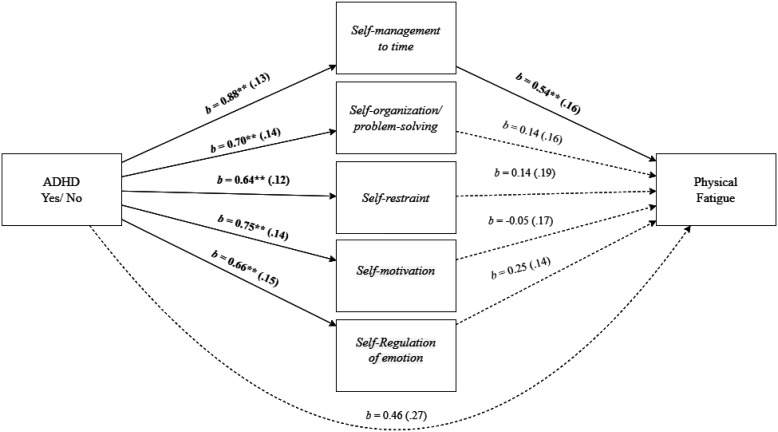
Mediation analysis for the relationship between employees' ADHD and physical fatigue, mediated by executive function deficit of self-management to time. Note: Standard errors are in parentheses. *p < 0.05, **p < 0.01.

As shown in Figure 4, employees' EF deficit of *Self-management to time* mediated the effect of employees' ADHD and *physical fatigue*, as indicated by a significant total indirect effect, *b* = 0.78, *SE* = 0.18, 95% *CI* [0.45, 1.18], specifically driven by the significant indirect effect of *Self-management to time, b* = 0.47, *SE* = 0.20, 95% *CI* [0.10, 0.88]. The direct effect did not remain significant, *b* = 0.46, *SE* = 0.27, 95% *CI* [-0.07, 0.99].

#### 3.3.2. Emotional exhaustion

**Figure 5.**
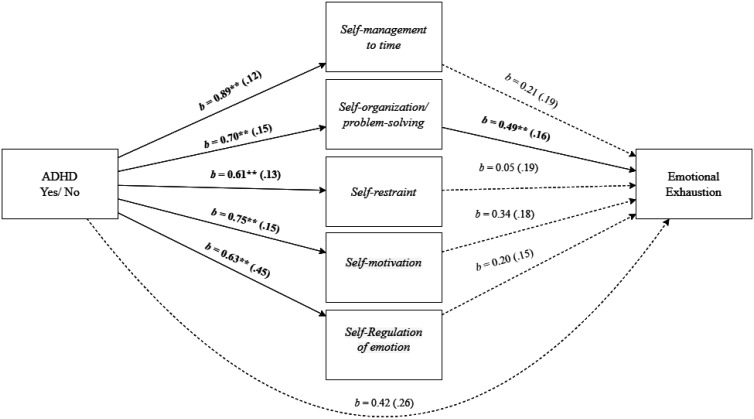
Mediation analysis for the relationship between employees' ADHD and emotional exhaustion, mediated by executive function deficit of self-organization/problem-solving. Note: Standard errors are in parentheses. *p < 0.05, **p < 0.01.

As shown in Figure 5, employees' EF deficit of *Self-organization/problem-solving* mediated the effect of employees' ADHD and *emotional exhaustion*, as indicated by a significant total indirect effect, *b* = 0.94, *SE* = 0.21, 95% *CI* [0.56, 1.38], specifically driven by the significant indirect effect of *Self-organization/problem-solving, b* = 0.34, *SE* = 0.14, 95% *CI* [0.11, 0.64]. The direct effect did not remain significant, *b* = 0.42, *SE* = 0.26, 95% *CI* [-0.11, 0.95].

#### 3.3.3. Cognitive weariness

**Figure 6.**
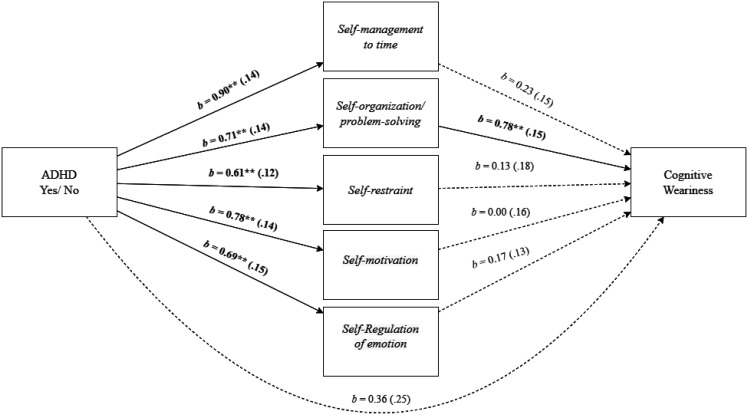
Mediation analysis for the relationship between employees' ADHD and cognitive weariness, mediated by executive function deficit of self-organization/problem-solving. Note: Standard errors are in parentheses. *p < 0.05, **p < 0.01.

As shown in Figure 6, employees' EF deficit of *Self-organization/problem-solving* mediated the effect of employees' ADHD and *cognitive weariness*, as indicated by a significant total indirect effect, *b* = 0.96, *SE* = 0.21, 95% *CI* [0.59, 1.40], specifically driven by the significant indirect effect of *Self-organization/problem-solving, b* = 0.55, *SE* = 0.16, 95% *CI* [0.28, 0.91]. The direct effect did not remain significant, *b* = 0.36, *SE* = 0.25, 95% *CI* [-0.14, 0.86].

### 3.4. Auxiliary analyses

We conducted additional mediation analyses while controlling for demographic variables: age, gender, seniority at work, and tenure. The indirect effect from ADHD to job burnout through EF deficits remained significant, *b* = 0.76, *SE* = 0.16, 95% *CI* [0.48, 1.09], the direct effect was significant, *b* = 0.51, *SE* = 0.20, 95% *CI* [0.12, 0.92]. The results remained the same when submitting each EF deficit separately as a mediator. As before, only the indirect effects through *self-management to time* and *self-organization/problem-solving* were significant, *b*s = 0.31, 0.30, *SE*s = 0.16, 0,12, 95% *CI*s [0.02, 0.63], [0.10, 0.57], respectively. The direct effect was significant *b* = 0.45, *SE* = 0.22, 95% *CI* [0.02, 0.88].

We also conducted mediation analyses using the aforementioned demographic covariate for the effects on each burnout subscale. When submitting physical fatigue as the dependent variable, only *self-management to time* had a significant indirect effect, *b* = 0.42, *SE* = 0.19, 95% *CI* [0.05, 0.80]. The direct effect was not significant *b* = 0.41, *SE* = 0.27, 95% *CI* [-0.12, 0.94]. With regard to emotional exhaustion as a dependent variable, *self-organization/problem-solving* had a significant indirect effect, *b* = 0.34, *SE* = 0.14, 95% *CI* [0.10, 0.65] as well as self-motivation, *b* = 0.25, *SE* = 0.14, 95% *CI* [0.01, 0.56]. The direct effect was not significant *b* = 0.43, *SE* = 0.27, 95% *CI* [-0.11, 0.97]. Finally, when submitting cognitive weariness as the dependent variable, only *self-organization/problem-solving* was a significant mediator, *b* = 0.53, *SE* = 0.16, 95% *CI* [0.24, 0.89]. The direct effect was not significant *b* = 0.41, *SE* = 0.26, 95% *CI* [-0.09, 0.92]. These results indicate that, overall, the demographic covariates did not change the conclusions.

## 4. General discussion

The present study aimed to investigate the interplay between ADHD, EF deficits, and job burnout. Our findings indicated significant correlations among study variables, with employees with ADHD reporting elevated levels of job burnout and EF deficits. The main effects analysis revealed a substantial impact of ADHD on both job burnout (consistent with H1) and EF deficits (consistent with H2). Mediation analysis confirmed that EF deficits mediated the relationship between ADHD and job burnout, supporting H3a. Further examination highlighted that consistent with H3b, the specific EF deficits of *Self-management to time* and *Self-organization/problem-solving* played a mediating role in the link between ADHD and job burnout.

The present work adds theoretically by delving into the intricate dynamics between ADHD, EF deficits, and job burnout. Our findings suggest that EF deficits might serve as an explanation for the relationship between ADHD and job burnout. Furthermore, the study disentangles the specific mechanisms by which these factors interact to impact job burnout. Specifically, the parallel mediation analyses indicated that *self-management to time* and *self-organization/problem-solving* were the most important mediators among EFs deficit for the relationship between ADHD and general job burnout as well as for its components -physical fatigue, emotional exhaustion, and cognitive weariness. Therefore, the present findings suggest that EF deficiency serves as an occupational challenge for employees with ADHD. It might also help explain turnover intentions and job-quitting, given that burnout serves as an antecedent of these two organizational outcomes [61]–[64].

In delving into the intricacies of our findings, it becomes crucial to provide a more nuanced account of the relationships observed. Specifically, our mediation analyses shed light on the role of EF deficits in the complex interplay between employees' ADHD and job burnout.

Firstly, when examining EF deficits as a mediator, we observed that *self-management to time* and *self-organization/problem-solving* played a pivotal role in mediating the relationship between ADHD and overall job burnout. This implies that a substantial portion of the association between ADHD and burnout can be attributed to deficits in these specific facets of EF. This finding aligns with previous literature on ADHD, which has suggested that challenges in time management and organizational skills could contribute to difficulties manifested in the workplace [31],[40]–[43].

Moving beyond the general mediation, a more detailed exploration of each burnout subscale provides a comprehensive understanding. Specifically, the mediation effect was evident in *physical fatigue*, *emotional exhaustion*, and *cognitive weariness*. For *physical fatigue*, the mediation was realized through *self-management to time*. This aligns with previous literature and findings suggesting that deficits in managing time efficiently may contribute to difficulties manifested in the workplace [32],[65]. The mediation was significant for emotional exhaustion and cognitive weariness through *self-organization/problem-solving*. This aligns with previous findings indicating that factors such as workload, incivility/conflicts/violence, negative work attitudes, and work-life conflict may contribute to emotional exhaustion [33]. These findings not only identify the occurrence of mediation but also conduct a detailed exploration, elucidating the specific facets of EF deficits and job burnout that are intricately linked. This precision enhances the empirical understanding of these relationships and offers valuable insights for implementing targeted interventions and support strategies in the workplace.

In conclusion, our study sheds new light on the intricate dynamics surrounding the impact of employees' ADHD on job burnout, emphasizing the pivotal role of EF deficits in this relationship. The findings underscore the significance of understanding how EF deficits contribute to the challenges faced by employees with ADHD. Practically, interventions targeting EF skills of *self-management to time* and *self-organization/problem-solving* may serve as preventive measures against job burnout among employees with ADHD. Furthermore, our findings suggest the necessity of workplace accommodations and support mechanisms tailored to address EF deficits among employees with ADHD. Recognizing and addressing these challenges within the professional environment may foster a more inclusive and supportive workplace, ultimately contributing to burnout prevention for individuals with ADHD.

## 5. Limitations & future research

Several limitations in our work should be addressed. Firstly, the reliance on self-report measures introduces the potential for common method bias, impacting the accuracy of responses. While self-reports are a common and valuable method, future studies could benefit from incorporating diverse data sources, such as observational assessments or reports from supervisors and colleagues, to provide a more comprehensive and objective understanding of the phenomena under investigation.

Second, while our findings provide valuable insights, our modest sample size may limit the generalizability of our results to broader populations. We acknowledge the importance of larger samples for robust statistical power and recommend that future studies consider more extensive participant pools. Furthermore, longitudinal investigations and diverse methodological approaches can provide a more comprehensive understanding of these dynamics, informing the development of targeted interventions and support strategies.

Our auxiliary analyses indicated that demographic variables, namely, age and gender, did not change the conclusions. Yet, our data is mute about other potential constructs that are known to be associated with ADHD and, therefore, might be relevent to the model. Examples include anxiety disorders [66],[67] and depression [68]. Future studies should replicate our study and control for such constructs.

Our findings highlight the critical need for tailored workplace interventions to address EF deficits in employees with ADHD, particularly focusing on *Self-management to time* and *Self-organization/problem-solving* as an effective strategy to alleviate job burnout. This emphasis is substantiated by prior research indicating that targeted interventions, including cognitive training programs, substantially improve the functioning of individuals facing EF deficits [69].

## 6. Conclusion

Our work aimed to investigate the relationships between employees' ADHD, EF deficits, and job burnout. By elucidating the mediating role of EF deficits in the link between employees' ADHD and burnout, our findings contribute to a deeper understanding of the mechanisms underlying the occupational challenges faced by employees with ADHD. Future research can build on our findings, further explore longitudinal designs, and incorporate objective measures to validate these findings. Ultimately, addressing EF deficits in employees with ADHD may pave the way for targeted interventions aimed at alleviating job burnout.

## Use of AI tools declaration

The authors declare no Artificial Intelligence (AI) tools have been used in the creation of this article.

## References

[1] American Psychiatric Association (2013). Diagnostic and statistical manual of mental disorders: DSM-5.

[2] Barkley RA (2002). Major life activity and health outcomes associated with attention-deficit/hyperactivity disorder. J Clin Psychiatry.

[3] Caye A, Swanson J, Thapar A (2016). Life span studies of ADHD—conceptual challenges and predictors of persistence and outcome. Curr Psychiatry Rep.

[4] Kooij JS, Buitelaar JK, FURER JW (2005). Internal and external validity of attention-deficit hyperactivity disorder in a population-based sample of adults. Psychol Med.

[5] Weiss G, Hechtman L, Milroy T (1985). Psychiatric status of hyperactives as adults: A controlled prospective 15-year follow-up of 63 hyperactive children. J Am Acad Child Psychiatry.

[6] Mannuzza S, Klein RG, Bessler A (1993). Adult outcome of hyperactive boys: Educational achievement, occupational rank, and psychiatric status. Arch Gen Psychiatry.

[7] Fuermaier AB, Tucha L, Butzbach M (2021). ADHD at the workplace: ADHD symptoms, diagnostic status, and work-related functioning. J Neural Transm.

[8] Secnik K, Swensen A, Lage MJ (2005). Comorbidities and costs of adult patients diagnosed with attention-deficit hyperactivity disorder. Pharmacoeconomics.

[9] Mannuzza S, Klein RG (2000). Long-term prognosis in attention-deficit/hyperactivity disorder. Child Adolesc Psychiatr Clin N Am.

[10] Murphy K, Barkley RA (1996). Attention deficit hyperactivity disorder adults: comorbidities and adaptive impairments. Compr Psychiatry.

[11] Biederman J, Petty C, Fried R (2006). Impact of psychometrically defined deficits of executive functioning in adults with attention deficit hyperactivity disorder. Am J Psychiatry.

[12] Frazier TW, Youngstrom EA, Glutting JJ (2007). ADHD and achievement: Meta-analysis of the child, adolescent, and adult literatures and a concomitant study with college students. J Learn Disabil.

[13] Kessler RC, Adler L, Ames M (2005). The World Health Organization Adult ADHD Self-Report Scale (ASRS): a short screening scale for use in the general population. Psychol Med.

[14] Oscarsson M, Nelson M, Rozental A (2022). Stress and work-related mental illness among working adults with ADHD: A qualitative study. BMC Psychiatry.

[15] Willcutt EG, Doyle AE, Nigg JT (2005). Validity of the executive function theory of attention-deficit/hyperactivity disorder: A meta-analytic review. Biol Psychiatry.

[16] Pihlaja M, Tuominen P, Peräkylä J (2022). Occupational burnout is linked with inefficient executive functioning, elevated average heart rate, and decreased physical activity in daily life-initial evidence from teaching professionals. Brain Sci.

[17] Shirom A, Quick JC, Tetrick LE (2003). Job-related burnout. Handbook of Occupational Health Psychology.

[18] Shirom A, Cooper C. L., Robertson I. T. (1989). Burnout in work organizations. International review of industrial and organizational psychology.

[19] Schaufeli WB, Buunk BP (2003). Burnout: An overview of 25 years of research and theorizing. The Handbook of Work and Health Psychology.

[20] Pines A, Aronson E (1988). Career burnout: Causes and cures.

[21] Shanafelt TD, Balch CM, Bechamps GJ (2009). Burnout and career satisfaction among American surgeons. Ann Surg.

[22] Soler JK, Yaman H, Esteva M (2008). Burnout in European family doctors: The EGPRN study. Fam Pract.

[23] Borritz M, Rugulies R, Christensen KB (2006). Burnout as a predictor of self-reported sickness absence among human service workers: Prospective findings from three year follow up of the PUMA study. Occup Environ Med.

[24] Duijts SF, Kant I, Swaen GM (2007). A meta-analysis of observational studies identifies predictors of sickness absence. J Clin Epidemiol.

[25] Maslach C, Schaufeli WB, Leiter MP (2001). Job burnout. Annu Rev Psychol.

[26] Shanafelt TD, Bradley KA, Wipf JE (2002). Burnout and self-reported patient care in an internal medicine residency program. Ann Intern Med.

[27] West CP, Huschka MM, Novotny PJ (2006). Association of perceived medical errors with resident distress and empathy: a prospective longitudinal study. JAMA.

[28] Shanafelt TD, Balch CM, Bechamps G (2010). Burnout and medical errors among American surgeons. Ann Surg.

[29] Leiter MP, Maslach C (2009). Nurse turnover: the mediating role of burnout. J Nurs Manag.

[30] Deligkaris P, Panagopoulou E, Montgomery AJ (2014). Job burnout and cognitive functioning: A systematic review. Work & Stress.

[31] Sarkis E (2014). Addressing attention-deficit/hyperactivity disorder in the workplace. Postgrad Med.

[32] Adamou M, Arif M, Asherson P (2013). Occupational issues of adults with ADHD. BMC Psychiatry.

[33] Lee RT, Seo B, Hladkyj S (2013). Correlates of physician burnout across regions and specialties: A meta-analysis. Hum Resour Health.

[34] Brattberg G (2006). PTSD and ADHD: Underlying factors in many cases of burnout. Stress Health.

[35] Chan T, Wang I, Ybarra O (2021). Leading and managing the workplace: The role of executive functions. Acad Manage Perspect.

[36] Barkley RA (2011). Barkley deficits in executive functioning scale (BDEFS).

[37] Kizony R, Demayo-Dayan T, Sinoff G (2011). Validation of the executive function route-finding task (EFRT) in people with mild cognitive impairment. OTJR (Thorofare N J).

[38] Thorell L, Holst Y, Chistiansen H (2017). Neuropsychological deficits in adults age 60 and above with attention deficit hyperactivity disorder. Eur Psychiatry.

[39] Bodalski EA, Knouse LE, Kovalev D (2019). Adult ADHD, emotion dysregulation, and functional outcomes: Examining the role of emotion regulation strategies. J Psychopathol Behav.

[40] Harpin VA (2005). The effect of ADHD on the life of an individual, their family, and community from preschool to adult life. Arch Dis Childhood.

[41] Murphy KR, Barkley RA (2007). Occupational functioning in adults with ADHD. ADHD Report.

[42] Nadeau KG (2005). Career choices and workplace challenges for individuals with ADHD. J Clin Psychol.

[43] Tominey EW, Tominey M, Bruyere SM (2001). Working effectively with people with attention deficit/hyperactivity disorder.

[44] Primich C, Iennaco J (2012). Diagnosing adult attention-deficit hyperactivity disorder: The importance of establishing daily life contexts for symptoms and impairments. J Psychiatr Ment Health Nurs.

[45] Toner M, O'Donoghue T, Houghton S (2006). Living in chaos and striving for control: How adults with attention deficit hyperactivity disorder deal with their disorder. Int J Disabil De Edu.

[46] Boonstra AM, Oosterlaan J, Sergeant JA (2005). Executive functioning in adult ADHD: A meta-analytic review. Psychol Med.

[47] Kamradt JM, Ullsperger JM, Nikolas MA (2014). Executive function assessment and adult attention-deficit/hyperactivity disorder: Tasks versus ratings on the Barkley deficits in executive functioning scale. Psychol Assess.

[48] Hervey AS, Epstein JN, Curry JF (2004). Neuropsychology of adults with attention-deficit/hyperactivity disorder: A meta-analytic review. Neuropsychology.

[49] Frazier TW, Demaree HA, Youngstrom EA (2004). Meta-analysis of intellectual and neuropsychological test performance in attention-deficit/hyperactivity disorder. Neuropsychology.

[50] Faul F, Erdfelder E, Lang A-G (2007). G* Power 3: A flexible statistical power analysis program for the social, behavioral, and biomedical sciences. Behav Res Methods.

[51] Faraone SV, Spencer TJ, Montano CB (2004). Attention-deficit/hyperactivity disorder in adults: A survey of current practice in psychiatry and primary care. Arch Intern Med.

[52] Wender PH, Wolf LE, Wasserstein J (2001). Adults with ADHD: An overview. Ann N Y Acad Sci.

[53] Ustun B, Adler LA, Rudin C (2017). The World Health Organization adult attention-deficit/hyperactivity disorder self-report screening scale for DSM-5. Jama Psychiatry.

[54] Barkley RS (2011). Barkley deficits in executive functioning scale (BDEFS).

[55] Lace JW, McGrath A, Merz ZC (2020). A factor analytic investigation of the Barkley deficits in executive functioning scale, short form. Curr Psychol.

[56] Flannery AJ, Luebbe AM, Becker SP (2017). Sluggish cognitive tempo is associated with poorer study skills, more executive functioning deficits, and greater impairment in college students. J Clin Psychol.

[57] Shirom A, Melamed S (2006). A comparison of the construct validity of two burnout measures in two groups of professionals. Int J Stress Manage.

[58] Gerber M, Colledge F, Mücke M (2018). Psychometric properties of the Shirom-Melamed Burnout Measure (SMBM) among adolescents: Results from three cross-sectional studies. BMC Psychiatry.

[59] Melamed S, Shirom A, Toker S (2006). Burnout and risk of type 2 diabetes: A prospective study of apparently healthy employed persons. Psychosom Med.

[60] Hayes AF (2017). Introduction to mediation, moderation, and conditional process analysis: A regression-based approach.

[61] Itzchakov G, Weinstein N, Cheshin A (2022). Learning to listen: Downstream effects of listening training on employees' relatedness, burnout, and turnover intentions. Hum Resour Man.

[62] Barthauer L, Kaucher P, Spurk D (2020). Burnout and career (un) sustainability: Looking into the Blackbox of burnout triggered career turnover intentions. J Vocat Behav.

[63] Fukui S, Wu W, Salyers MP (2019). Impact of supervisory support on turnover intention: The mediating role of burnout and job satisfaction in a longitudinal study. Adm Policy Ment Health.

[64] Dunford BB, Shipp AJ, Boss RW (2012). Is burnout static or dynamic? A career transition perspective of employee burnout trajectories. J Appl Psychol.

[65] Holst Y, Thorell LB (2019). Functional impairments among adults with ADHD: a comparison with adults with other psychiatric disorders and links to executive deficits. Appl Neuropsychol Adult.

[66] D'Agati E, Curatolo P, Mazzone L (2019). Comorbidity between ADHD and anxiety disorders across the lifespan. Int J Psychiatry Clin Pract.

[67] Koyuncu A, Ayan T, İnce Guliyev E (2022). ADHD and anxiety disorder comorbidity in children and adults: Diagnostic and therapeutic challenges. Curr Psychiatry Rep.

[68] Riglin L, Leppert B, Dardani C (2021). ADHD and depression: Investigating a causal explanation. Psychol Med.

[69] Zelazo PD, Blair CB, Willoughby MT (2016). Executive function: Implications for education. NCER 2017–2000.

